# Fucoxanthin Prevents 6-OHDA-Induced Neurotoxicity by Targeting Keap1

**DOI:** 10.1155/2021/6688708

**Published:** 2021-03-11

**Authors:** Wei Wu, Hui Han, Jingwangwei Liu, Min Tang, Xiaoyu Wu, Xiaojun Cao, Tiantian Zhao, Yujia Lu, Tingting Niu, Juanjuan Chen, Haimin Chen

**Affiliations:** State Key Laboratory for Managing Biotic and Chemical Threats to the Quality and Safety of Agro-products, Ningbo University, Ningbo, Zhejiang 315211, China

## Abstract

As the most abundant marine carotenoid extracted from seaweeds, fucoxanthin (FUC) is considered to have excellent neuroprotective activity. However, the target of FUC for its neuroprotective properties remains largely unclear. Oxidative stress is one of the initiating factors causing neuronal cell loss and necrosis, and it is also an important inducement of Parkinson's disease (PD). In the present study, the neuroprotective effect of FUC was assessed using a 6-hydroxydopamine- (6-OHDA-) induced neurotoxicity model. FUC suppressed 6-OHDA-induced accumulation of intracellular ROS, the disruption of mitochondrial membrane potential, and cell apoptosis through the Nrf2-ARE pathway. Keap1 as a repressor of Nrf2 can regulate the activity of Nrf2. Here, the biolayer interferometry (BLI) assay demonstrated that FUC specifically targeted Keap1 and inhibited the interaction between Keap1 and Nrf2. FUC bound to the hydrophobic region of Keap1 pocket and formed hydrogen bonding interactions with Arg^415^ and Tyr^525^. Besides, it also dose-dependently upregulated the expressions of antioxidant enzymes, such as nicotinamide heme oxygenase-1, glutamate-cysteine ligase modifier subunit, and glutamate-cysteine ligase catalytic subunit, in 6-OHDA-induced PC12 cells. In 6-OHDA-exposed zebrafish, FUC pretreatment significantly increased the total swimming distance of zebrafish larvae and improved the granular region of the brain tissue damage. These results suggested that FUC could protect the neuronal cells against 6-OHDA-induced injury via targeting Keap1.

## 1. Introduction

Parkinson's disease (PD) is a progressive neurodegenerative disease, which is characterized by tremor, rigidity, akinesia, and postural instability [1]. Based on the Global Burden of Disease study, neurodegenerative disorders are currently the leading cause of disability worldwide, and the incidence of PD has been rapidly increasing. From 1990 to 2015, the global number of PD patients is increased by 118% to 6.2 million [2]. Therefore, PD has become a major threat to global public health.

The key pathogenic factors of PD include neuronal apoptosis in the substantia nigra, excessive oxidative stress, immune abnormality, mitochondrial dysfunction, and excitatory toxicity [3]. Recent investigations have shown that the dramatic reduction of dopamine (DA) content in the striatum and the dysfunction of dopaminergic neurons in the substantia nigra of the middle brain contribute to the progression of PD [4]. However, the currently available treatments for PD mainly include administration of levodopa or supplementation of DA receptor activators [4]. Although these strategies can alleviate PD symptoms to some extent, their long-term use will cause adverse reactions, such as symptom fluctuations, switching phenomena, and movement disorders. Moreover, some studies have shown that a high concentration of DA in the cytoplasm can produce cytotoxicity and lead to the death of DA neurons [5]. The reason may be attributed to the fact that DA can be oxidized to 3,4-dihydroxyphenylacetaldehyde (DOPAL) and hydrogen peroxide by the monoamine oxidase in the cell, and DOPAL itself is oxidized to produce reactive oxygen species (ROS), or DA can generate hydroxyl radical, superoxide anion radical, and hydrogen peroxide by autooxidation, leading to oxidative damage to cells [6]. Therefore, compounds with inhibitory effects on intracellular oxidative stress could be used to prevent the progression of PD.

Researchers have found many antioxidants to overcome the oxidative stress in dopaminergic neurons. Taghizadeh et al. have reported that a VE-rich diet can reduce the risk of PD [7]. Coenzyme Q10 delays the course of PD and can improve certain clinical symptoms of PD [8]. Ginsenoside Rg1 can not only ameliorate the symptoms of PD but also reduce the side effects of levodopa [9, 10]. At present, it has been well recognized that pyrroloquinoline quinone (PQQ) can prevent PD to preserve DA neurons by protecting mitochondrial complex I and scavenging oxygen free radicals [11].

As the most abundant carotenoid extracted from seaweeds, fucoxanthin (FUC) accounts for more than 10% of the total natural carotenoid [12]. Because of its unique structure, FUC exhibits a variety of pharmacological properties, such as reducing oxidative stress and repressing inflammatory reactions [13]. Besides, FUC also has significant neuroprotective effects, which are related to the etiology of PD. The safety of FUC is also confirmed. Even if the ingestion reaches 200 mg/kg·day, there is still no teratogenicity in vivo, and it is considered a safe antioxidant [14]. So far, the molecular mechanisms underlying the antioxidant effect of FUC have been preliminarily clarified [15], while the target of FUC for its antioxidant properties remains largely unexplored.

Therefore, we mainly aimed to identify the molecular target of FUC as well as the underlying mechanism of its neuroprotective effect. We determined the effects of FUC on the conformation of the Keap1/Nrf2 complex by immunoprecipitation (IP) assay. In addition, we assessed the effects of FUC on the binding between Keap1 protein and FUC and explored the latent protective effect of FUC against 6-OHDA-triggered neurotoxicity in PC12 cells and zebrafish. Taken together, our study identified a new Keap1 specific inhibitor, FUC, which could be used as a potential candidate in the prevention of neurodegenerative diseases.

## 2. Materials and Methods

### 2.1. Cell Culture and FUC Preparation

The PC12 cell line was purchased from China Center for Type Culture Collection (CTCC, Wuhan, Hubei, China) and maintained in Roswell Park Memorial Institute 1640 medium (Gibco BRL, Grand Island, NY, USA) consisting of 10% heat-inactivated horse serum (Gibco BRL, Grand Island, NY, USA) and 10% fetal bovine serum (Gibco BRL, Grand Island, NY, USA) at 37°C in a humidified atmosphere containing 5% CO_2_. FUC (Biopurify, Chengdu, Sichuan, China) was dissolved in dimethyl sulfoxide (DMSO, Sigma, St. Louis, MO, USA) and then diluted to desired concentrations.

### 2.2. Co-IP Assay

PC12 cells were cultivated for 24 h in the absence or presence of FUC at different concentrations, followed by lysis in an extraction buffer. The cell lysates were centrifuged at 12,000 rpm for 10 min at 4°C. Subsequently, 500 *μ*g of purified protein was mixed with anti-Keap1 antibody (Cell Signaling Technology, Danvers, MA, USA), and the mixture was mildly agitated at 4°C overnight. The immunocomplex was harvested with protein A+G agarose, and the precipitates were rinsed with precooled (4°C) phosphate-buffered saline (PBS) three times. Next, the samples were disassociated by boiling to release the proteins, followed by Western blotting analysis using the anti-Nrf2 antibody (1 : 1,000, Abcam Inc., Cambridge, MA, USA).

### 2.3. Protein Expression and Purification of Keap1 Mutation Protein

The cDNA of rhKeap1 mutation (rhKeap1^R415A+Y525A+^; residue 321-609 aa, PDB ID: 4IFN) was synthesized by NovoPro Bioscience Inc. (Shanghai, China). The cDNA fragment was then ligated into a pET28a vector (NovoPro Bioscience Inc., Shanghai, China). The DNA series of rhKeap1^R415A+Y525A+^ are listed in Supplemental Material (available here). The rhKeap1^R415A+Y525A+^-pET28a plasmid was transformed into *E. coli* BL21 (DE3). The protein expression was induced by 1 mM isopropyl-b-D-(-) thiogalactoside at 28°C for 8 h. The inclusion bodies were collected and dissolved with 50 mM Tris-HCl, 0.6 M NaCl, and 8 M urea (pH 8.0) and stored at room temperature for 12 h.

Subsequently, any residual insoluble matter was removed by centrifuging the samples at 12,000 rpm for 0.5 h, and the supernatant was filtered through a 0.22 *μ*m filter (EMD Millipore, Billerica, MA). The supernatant was loaded onto Ni-IDA Sepharose 6 Fast Flow (General Electric, Fairfield, CT) according to the manufacturer's instructions. Refolding of rhKeap1^R415A+Y525A+^ peptides was conducted by gradient dialysis against 6–0.5 M urea in 50 mM Tris-HCl and 0.6 mM NaCl (pH 8.0) at 4°C. The concentration of purified protein was determined using the Bradford method.

### 2.4. FortéBio Octet System Assay for Binding Affinity between Keap1 Protein or Keap1 Mutation Protein or Nrf2 Protein and FUC

The interaction between FUC and human Keap1 protein (Sino Biological, Beijing, China), human Nrf2 protein (NovoPro Bioscience Inc., Shanghai, China), or rhKeap1^R415A+Y525A+^ protein was tested by biolayer interferometry (BLI) assay on an Octet K2 system (FortéBio, Fremont, CA, USA). For the binding affinity analysis, the sensors were loaded with biotinylated Keap1 or Nrf2 or rhKeap1^R415A+Y525A+^ protein for 15 min and then quenched in 100 *μ*M biotin for 1 min. FUC of various concentrations was prepared in PBS (pH 7.4). The immobilized sensor was dipped into the test FUC solution for association and then returned to the blocking solution (8 mM Na_2_HPO_4_, 0.136 M NaCl, 2 mM KH_2_PO_4_, 2.6 mM KCl, and 0.05% (*v*/*v*) Tween-20, 5% DMSO pH 7.4) for dissociation. Keap1, Nrf2, or rhKeap1^R415A+Y525A+^ protein was used as the positive control. Data were assessed using the FortéBio analysis software (Version: 9.0.0.10). Regression analysis was adopted to determine the kinetics parameters (*K*_on_ and *K*_off_) and affinity constants (KD) from a nonlinear global fit.

To assess the interference effect of FUC on Nrf2-Keap1 binding, 266 nM Nrf2 and FUC of different concentrations were mixed, and the mixture was used to interact with Keap1 protein. The sensors were loaded with biotinylated Keap1 protein, and the immobilized sensor was dipped into the mixture containing FUC and Nrf2 protein for the association and then returned to the blocking solution for dissociation. Keap1 protein was employed as the positive control.

### 2.5. Docking of FUC to the Keap1 Structure Model

Molecular docking of the FUC-Keap1 complex was conducted by AutoDock Vina 1.1.2. The crystal structure of Keap1 (PDB ID: 4IFN) was downloaded from the Protein Data Bank (http://www.rcsb.org). The structure of FUC was obtained from the PubChem database (https://pubchem.ncbi.nlm.nih.gov/). According to the ligand position, the final coordinates and box size of Vina molecular docking were determined as follows: center_*x* = 46.768, center_*y* = 6.017, center_*z* = 14.345, size_*x* = 30, size_*y* = 30, and size_*z* = 30. To increase the calculation accuracy, the exhaustiveness parameter was set to 20. Unless otherwise specified, the default values were used for all parameters. Finally, docked conformations were clustered within the tolerance of 1 Å root-mean-square deviation. The docking and binding mode was analyzed by selecting the conformation with the lowest docking and binding energy, and the diagrams were prepared by Discovery Studio.

### 2.6. Western Blotting Analysis

The PC12 cells were seeded into 6-well plates and cultured for 12 h until 60% confluence. Subsequently, cells were pretreated with or without FUC of indicated concentrations for 2 h, followed by the addition of 250 *μ*M 6-OHDA (Aladdin, Shanghai, China) and incubation for 24 h. Concentration and exposure duration were selected based on previous research using the same reagents in PC12 cells [16]. As previously described [17], cultured cells were lysed in RIPA buffer (Solarbio, Beijing, China). Nuclear extracts were prepared by using the NE-PER nuclear and cytoplasmic extraction kit (Rockford, IL, USA) according to the manufacturer's protocol. The supernatants were collected after centrifugation (4°C, 11,492 × g, 15 min). Proteins were quantified using a BCA Protein Assay Kit (Thermo Fisher Scientific, Shanghai, China). Equal amounts of proteins were subjected to sodium dodecyl sulphate-polyacrylamide gel electrophoresis (SDS-PAGE) using 10% gels and then electrotransferred onto PVDF membranes. The blots were incubated with commercial primary antibodies against Keap1 (1 : 1,000, Cell Signaling, Danvers, MA, USA), Nrf2 (1 : 1,000, Abcam Inc., Cambridge, MA, USA), nicotinamide heme oxygenase-1 (HO-1, 1 : 10,000, Abcam Inc., Cambridge, MA, USA), glutamate-cysteine ligase catalytic subunit (GCLC, 1 : 1,000, Boster Biological Technology Co., Ltd., Wuhan, Hubei, China), glutamate-cysteine ligase modifier subunit (GCLM, 1 : 1,000, ABclonal, Wuhan, Hubei, China), histone (1 : 1,000, Cell Signaling, Danvers, MA, USA), and *β*-actin (1 : 1,000, Santa Cruz Biotechnology, CA, USA). Next, the blots were incubated with appropriate horseradish peroxidase- (HRP-) conjugated secondary antibodies (mouse anti-rabbit IgG, 1 : 2,000, goat anti-mouse IgG, 1 : 8,000, Santa Cruz Biotechnology, CA, USA) at room temperature for 1 h and then washed with TBST three times. The immunoreactive bands were detected by Western Bright ECL-HRP Substrate (Advansta Inc., Menlo Park, CA, USA). The ImageJ software was adopted to analyze the band intensity, and *β*-actin or histone served as a loading control.

### 2.7. Transient Transfection and Luciferase Reporter Assays

To examine the effect of FUC on ARE activation, PC12 cells were transiently cotransfected with 1 *μ*g of firefly luciferase reporter plasmid p-ARE-Luc (Clontech Laboratories, Palo Alto, CA, USA) and 0.1 *μ*g p-RL using X-tremeGENE HP DNA Transfection Reagent (Roche) according to the manufacturer's instructions. At 24 h after transfection, the cells were treated with different concentrations of FUC for 18 h in 6-OHDA-induced PC12 cells. The Dual-Glo Luciferase Assay System (Promega, Madison, WI, USA) was used to measure the firefly and Renilla luciferase activity in cell lysates. All experiments were repeated three times, and the luciferase activity was calculated and normalized to Renilla luciferase activity.

### 2.8. Intracellular ROS Measurements

The level of intracellular ROS was assessed with 5-(and-6)-carboxy-2,7-dichlorodihydrofluorescein diacetate (DCFH-DA, Sigma, St. Louis, MO, USA) as previously reported [18]. Briefly, treated cells were washed once with ice-cold PBS, followed by incubation with 10 *μ*M carboxy-H2DCF-DA at 37°C for 10 min. Cells were then washed once with ice-cold PBS and analyzed using a Beckman Gallios Flow Cytometer (Beckman Counter, Inc., Brea, CA, USA) at an excitation wavelength of 488 nm and an emission wavelength of 525 nm. Unless otherwise indicated, the fluorescence intensity in untreated PC12 cells was used as a control group.

### 2.9. Flow Cytometric Analysis of Cell Apoptosis

Cell apoptosis was assessed by using Annexin V and PI. Briefly, the treated cells were washed with PBS and resuspended in the binding buffer containing Annexin V-FITC and PI, followed by the incubation for 15 min in the dark. Then, the cells were detected by the Beckman Gallios Flow Cytometer (Beckman Counter, Inc., Brea, CA, USA). The excitation and emission wavelengths of FITC were 488 nm and 530 nm, respectively. The excitation and emission wavelengths of PI were 488 nm and 630 nm, respectively.

### 2.10. Measurement of Mitochondrial Membrane Potential

JC-1 (5,5′,6,6′-tetrachloro-1,1′,3,3′-tetraethyl-imidacarbocyanine iodide) staining was adopted to determine the mitochondrial membrane potential. Briefly, the cells treated as indicated were resuspended in 0.5 mL of cell culture medium and then mixed with 1 mL JC-1 staining solution. The mixture was incubated at 37°C for 20 min and then washed twice with JC-1 staining buffer. Images were acquired using a fluorescence confocal microscope (Zeiss LSM 880, Oberkochen, Germany).

### 2.11. Locomotion Behavioral Test of Zebrafish

The wild-type zebrafish of AB strain were obtained from Shanghai Fish Bio Co., Ltd. (Shanghai, China), and all animal-related experiments were approved by the Ningbo University Animal Research Advisory Committee. Zebrafish larvae of 3 days post fertilization (dpf) were put into 6-well plates (10 larvae/well), followed by exposure to FUC of various concentrations. After 2 h, 250 *μ*M 6-OHDA was added, followed by a further incubation of 4 days. Drug concentrations and exposure protocols were selected based on previous research using the same reagents in larval zebrafish [19, 20]. After incubation, larvae were transferred (using disposable 2 mL transfer pipets) into new 6-well plates containing regular fish water. The larvae were allowed to accommodate the new environment for 30 min. Subsequently, the swimming pattern and total distance traveled of each fish during 10 min were recorded. Zebrafish behavior was analyzed using an automated video tracking system (Viewpoint, ZebraLab, Life Sciences).

### 2.12. Histopathological Assessment

Treated zebrafish larvae at 7 dpf were fixed in 4% paraformaldehyde for 24 h, embedded with paraffin, and excised into 5 *μ*m sections, followed by hematoxylin and eosin (H&E) staining.

### 2.13. Detection of ROS in Zebrafish

Treated zebrafish larvae at 7 dpf were transferred into new 6-well plates (10 fish/well) containing regular fish water. Subsequently, the larvae were exposed to DCFH-DA (10 *μ*M) at 27°C for 60 min. Before the imaging, zebrafish were washed three times with embryo medium [21]. Fluorescence images of zebrafish were acquired by an inverted fluorescence microscope (NIKON TI-S, Tokyo, Japan).

### 2.14. Real-Time Quantitative PCR (RT-qPCR)

Treated zebrafish larvae at 7 dpf and PC12 cells were harvested and homogenized, and total RNA was purified using RNA-Solv® Reagent (Omega Bio-tek Inc., Norcross, GA, USA). Briefly, the first-strand cDNA was synthesized using 1 *μ*g of total RNA. RT-qPCR was performed on a LightCycler 96 Real-Time PCR System (Roche, Switzerland) using SYER-Green I. Nine pairs of specific primers were designed as follows [22–24]: for zebrafish: Keap1 forward 5′-TGTGATCTGGTTCTGCATGTC-3′ and reverse 5′-ACTCCTTGAAGTTGCTGGTG-3′; Nrf2 forward 5′-CTGCTGTCACTCCCAGAGTT-3′ and reverse 5′-GCCGTAGTTTTGGGTTGGTG-3′; HO-1 forward 5′-AAGAGCTGGACAGAAACGCA-3′ and reverse 5′-AGAAGTGCTCCAAGTCCTGC-3′; GCLC forward 5′-CTCCTCACAGTCACGGCATT-3′ and reverse 5′-TGAATGGAGACGGGGTGTTG-3′; GCLM forward 5′-AAGCCAGACACTGACACACC-3′ and reverse 5′-ATCTGGAGGCATCACACAGC-3′; *β*-actin forward 5′-CACTGAGGCTCCCCTGAATC-3′ and reverse 5′-GGGTCACACCATCACCAGAG-3′; for PC12 cells: Nrf2 forward 5′-TGGTGGTTTGCTACGACG-3′ and reverse 5′-CTCCAGAACTCC AGGCGG-3′; *β*-actin forward 5′-ATGGCAACTGTCCCTGAACT-3′ and reverse 5′-GTCATC ATCCCACGAGTCAC-3′. All primers were synthesized by Invitrogen (Shanghai, China), and *β*-actin was adopted as a housekeeping gene.

### 2.15. Statistical Analysis

Data were assessed using the SPSS software, version 16.0 (SPSS Inc., Chicago, IL, USA). The results were expressed as means ± SD. All results were assessed using one-way ANOVA with the Tukey–Kramer posttest and unpaired *t*-test. *P* < 0.05 was considered statistically significant. *P* < 0.01 was considered statistically highly significant.

## 3. Results and Discussion

### 3.1. FUC Blocks the Interaction between Keap1 and Nrf2 and Directly Binds to Keap1 Protein

**Figure 1:**
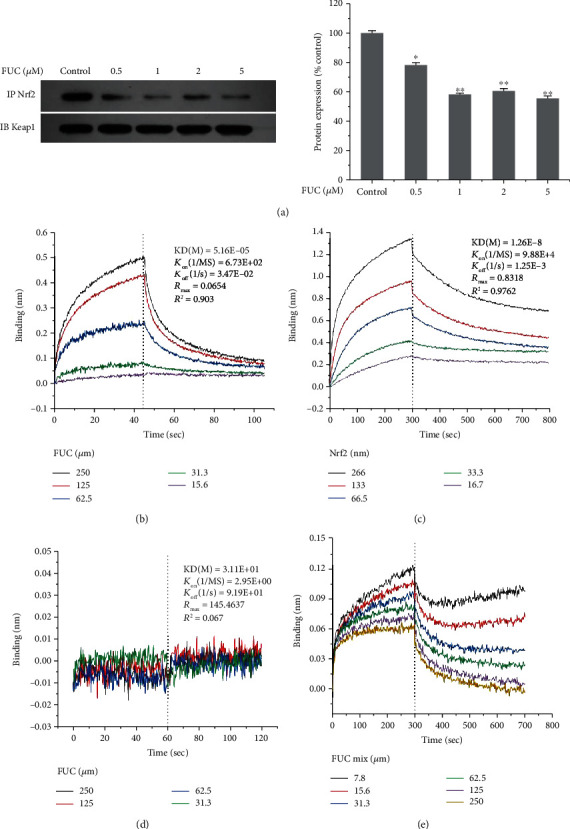
Inhibitory effect of FUC on Nrf2-Keap1 binding. (a) The effect of FUC on the conformation of Keap1/Nrf2 complex by IP assay. PC12 cells were pretreated with different concentrations of FUC for 2 h and then exposed to 250 *μ*M 6-OHDA for 24 h. Total protein was extracted and incubated with beads and anti-Keap1 antibody overnight at 4°C. The immunoprecipitated proteins were subjected to SDS-PAGE and detected using anti-Nrf2 antibody (IP), and the total Keap1 protein was detected by anti-Keap1 (IB). ^∗^*P* < 0.05 and ^∗∗^*P* < 0.01 versus the control group. (b) Kinetic binding analysis of FUC and Keap1 protein. (c) Kinetic binding analysis of Nrf2 protein and Keap1 protein. (d) Kinetic binding analysis of FUC and Nrf2 protein. (e) FUC interfered with Nrf2-Keap1 binding.

We first determined the effect of FUC on the conformation of the Keap1/Nrf2 complex by IP assay.  Figure 1(a) shows that the amount of Keap1/Nrf2 complex was decreased by 21.73%–44.93% in FUC-treated PC12 cells compared with the control group. We further tested how FUC could interfere with the conformation of Keap1/Nrf2 complex. The direct interaction between FUC and Keap1 protein was determined using BLI assay.  Figure 1(b) shows that FUC bound to Keap1 protein in a dose-dependent manner, with constant of KD = 5.16*E* − 5, constant of *K*_on_ = 6.73*E* + 2, and constant of *K*_off_ = 3.47*E* − 2. At high concentrations, the probe thickness could reach 0.5 nm. However, the interaction between Keap1 and Nrf2 showed that the binding constants (*K*_on_), dissociation constants (*K*_off_), and affinity constants (KD) were 9.88*E* + 4, 1.25*E* − 3, and 1.26*E* − 8, respectively. The KD values reflected that the binding affinity of Keap1 to Nrf2 protein was quite high (Figure 1(c)). There was no interaction between FUC and Nrf2 protein (Figure 1(d)). To further verify whether FUC could competitively inhibit the Nrf2/Keap1 binding, Nrf2 protein was mixed with different concentrations of FUC, and the mixture was used to interact with Keap1. The result showed that FUC could significantly decrease the binding affinity of Nrf2 to Keap1 protein in a dose-dependent manner (Figure 1(e)). These data indicated that FUC could suppress the binding of Keap1 to Nrf2 to some extent.

### 3.2. FUC Interacts with Arg415 and Tyr525 Residues in Keap1 Protein Pocket

**Figure 2:**
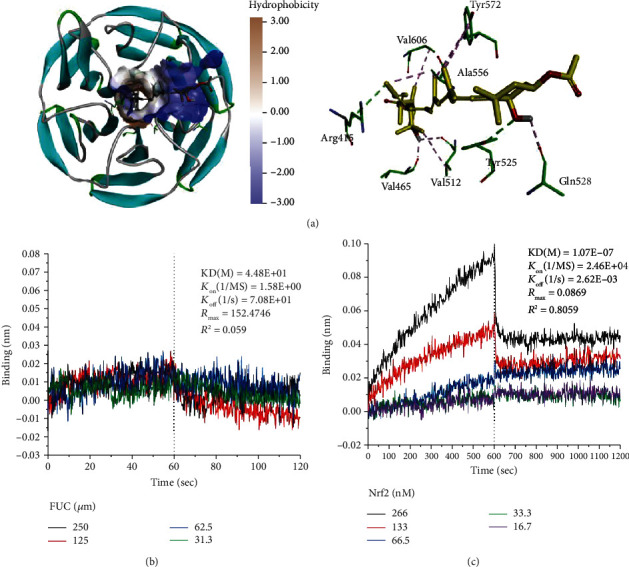
Binding mode of FUC to Keap1 protein. (a) Molecular docking of FUC with Keap1 (PDB ID: 4IFN). The green dotted lines are hydrogen bonds, and the purple dotted lines are hydrophobic bonds. (b) Kinetic binding analysis of FUC and Keap1 mutation protein, rhKeap1^R415A+Y525A+^. (c) Kinetic binding analysis of Nrf2 and Keap1 mutation protein, rhKeap1^R415A+Y525A+^.

A molecular simulation of the FUC-Keap1 complex was performed to further predict the underlying binding mode of FUC to Keap1 protein.  Figure 2(a) shows that FUC was fitted into the hydrophobic pocket of Keap1, and FUC interacted with several residues, including Arg^415^, Tyr^525^, Tyr^572^, Ala^556^, Val^606^, Val^512^, Val^465^, and Gln^528^, in a most energetically favorable configuration. Among these residues, two amino residues Arg^415^ and Tyr^525^ were the most probable candidates to form hydrogen bonds with FUC (Figure 2(a)). Therefore, to reveal the effect of Arg^415^ and Tyr^525^ on FUC binding to Keap1, an rhKeap1 mutation, rhKeap1^R415A+Y525A+^, was prepared. BLI assay indicated that the binding constants (*K*_on_), dissociation constants (*K*_off_), and affinity constants (KD) of the interaction between FUC and rhKeap1^R415A+Y525A+^ were 1.58*E* + 0, 7.08*E* + 1, and 4.48*E* + 1, respectively. The KD value reflected that FUC no longer bound to rhKeap1^R415A+Y525A+^ mutation protein (Figure 2(b)). In addition, KD between rhKeap1^R415A+Y525A+^ and Nrf2 was also decreased to 1.07*E* − 7, which was reduced by 8.49-fold compared with the binding affinity of Keap1 to Nrf2 protein (Figure 2(c)). These results indicated that Arg^415^ and Tyr^525^ were related to the binding of Nrf2 and Keap1, and these two residues were also the binding sites of FUC.

### 3.3. FUC Prevents 6-OHDA-Induced Decrease of Antioxidant Enzyme Level in PC12 Cells

**Figure 3:**
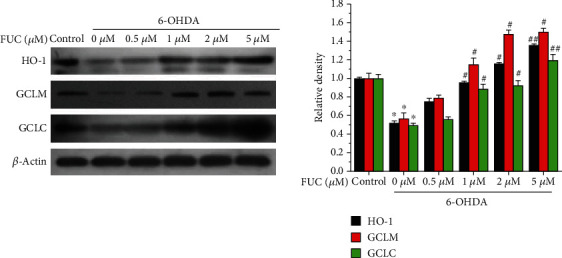
FUC downregulates 6-OHDA-induced expressions of antioxidant enzymes. ^∗^*P* < 0.05 versus the control group; ^#^*P* < 0.05 and ^##^*P* < 0.01 versus the 6-OHDA alone treatment group.

FUC is considered to have excellent antioxidative activity [25, 26]. Therefore, we examined the effect of FUC on the expressions of Keap1 downstream antioxidant enzymes in 6-OHDA-challenged PC12 cells by Western blotting analysis. We found that 6-OHDA significantly reduced the expressions of HO-1, GCLM, and GCLC (Figure 3). Compared with the control group, the expressions of HO-1, GCLM, and GCLC were reduced by 47.77%, 43.24%, and 50.25%, respectively, in the 6-OHDA treatment group. With FUC pretreatment, the expressions of these antioxidants were increased in a dose-dependent manner. Compared with the 6-OHDA alone treatment group, 5 *μ*M FUC significantly increased the expressions of HO-1, GCLM, and GCLC by 2.02-fold, 2.39-fold, and 2.39-fold, respectively (*P* < 0.05, Figure 3).

### 3.4. FUC Reverses the Inhibition of 6-OHDA on Keap1/Nrf2-ARE by Interacting with Keap1 in PC12 Cells

**Figure 4:**
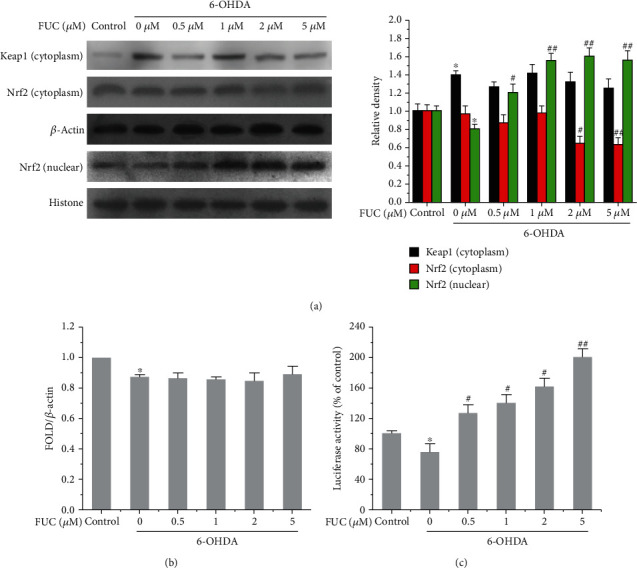
FUC activates the Nrf2/Keap1-ARE pathway in 6-OHDA-induced PC12 cells. (a) FUC increased the nuclear accumulation of Nrf2 in 6-OHDA-induced PC12 cells. (b) Effect of FUC on the expression of Nrf2 at the mRNA level in PC12 cells. Total RNA was extracted, and the expression of Nrf2 at the mRNA level was quantified with RT-qPCR and normalized to *β*-actin. (c) FUC increased the ARE activation in 6-OHDA-induced PC12 cells. PC12 cells were transiently transfected with p-ARE-Luc reporter plasmid and then treated with different concentrations of FUC for 18 h in 6-OHDA-induced PC12 cells. Firefly and Renilla luciferase activity was detected, and the fold induction was calculated by normalizing to the Renilla luciferase activity. ^∗^*P* < 0.05 versus the control group; ^#^*P* < 0.05 and ^##^*P* < 0.01 versus the 6-OHDA alone treatment group.

Keap1/Nrf2 is the major pathway that regulates phase II antioxidant responses, which can protect cells from oxidative stress-induced cytotoxicity [25, 26]. As shown in Figure 4(a), the expression of Keap1 was significantly increased, and intranuclear Nrf2 was significantly decreased with the exposure of 6-OHDA compared with controls (*P* < 0.05). However, we found that FUC pretreatment caused a decrease in the level of Nrf2 protein in the cytoplasm and significantly increased the accumulation of Nrf2 protein in the nucleus in 6-OHDA-induced PC12 cells. Notably, 2 *μ*M FUC significantly increased the accumulation of nuclear Nrf2 protein by 1.63-fold and significantly reduced the level of cytoplasmic Nrf2 by 36.17% in 6-OHDA-induced PC12 cells compared with the 6-OHDA treatment group (*P* < 0.05, Figure 4(a)). Although 6-OHDA treatment decreased the total mRNA level of Nrf2 and increased the expression of Keap1 protein (*P* < 0.05), with FUC pretreatment, the expressions of Keap1 protein and the total mRNA level of Nrf2 were not significantly changed in 6-OHDA-induced PC12 cells compared with the 6-OHDA treatment group (*P* > 0.05, Figures 4(a) and 4(b)).

In addition, we examined the induction of a luciferase reporter gene containing Nrf2-dependent antioxidant response element (ARE-Luc). As shown in Figure 4(c), 6-OHDA treatment inhibited the activity of ARE-luciferase compared with the control group (*P* < 0.05). However, FUC pretreatment significantly increased the activity of ARE-luciferase in 6-OHDA-induced PC12 cells compared with the 6-OHDA treatment group (*P* < 0.05).

### 3.5. FUC Protects PC12 Cells from 6-OHDA-Induced Damage

**Figure 5:**
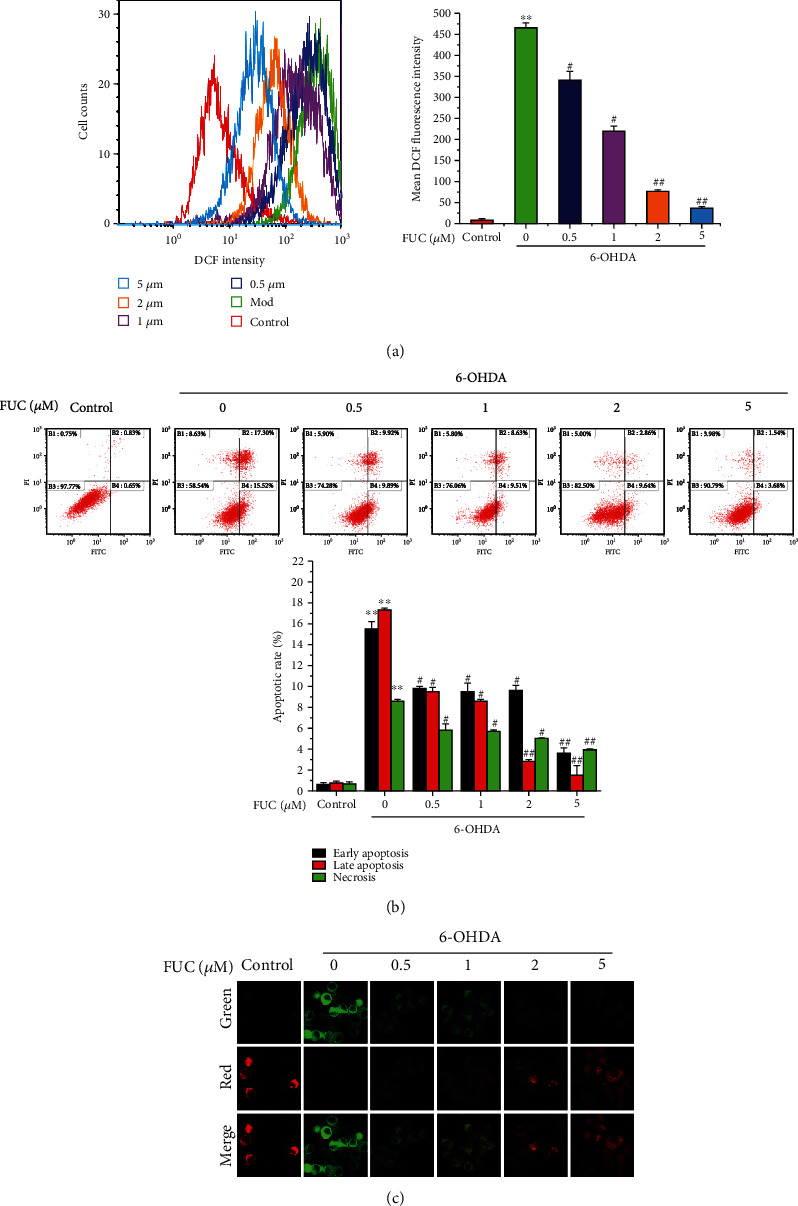
FUC prevents 6-OHDA-induced damage of PC12 cells. (a) The effect of FUC on 6-OHDA-induced cellular ROS level in PC12 cells. The PC12 cells were treated with DCFH-DA (10 *μ*M), and the ROS level was detected by a flow cytometer with an excitation wavelength of 488 nm and an emission wavelength of 525 nm. (b) The effect of FUC on 6-OHDA-induced apoptosis. The treated cells were incubated with Annexin V-FITC and PI and then detected by a flow cytometer. The excitation and emission wavelengths of FITC were 488 nm and 530 nm, respectively. The excitation and emission wavelengths of PI were 488 nm and 630 nm, respectively. (c) The effect of FUC on mitochondrial membrane in 6-OHDA-exposed PC12 cells. PC12 cells were pretreated with FUC for 2 h and then exposed to 6-OHDA for 24 h. Subsequently, the cells were then treated with JC-1 probe and imaged by a confocal microscope. ^∗∗^*P* < 0.01 versus the control group. ^#^*P* < 0.05 and ^##^*P* < 0.01 versus the 6-OHDA alone treatment group.

The level of intracellular ROS was detected by DCFH-DA. After the cells were incubated with 250 *μ*M 6-OHDA for 24 h, the level of intracellular ROS was increased by 53.34-fold compared with the control group (*P* < 0.01, Figure 5(a)). However, compared with the 6-OHDA alone treatment group, FUC pretreatment significantly reduced the level of ROS by 26.64–92.17% (*P* < 0.01, Figure 5(a)).

Apoptosis was determined using Annexin V-FITC/PI.  Figure 5(b) shows that after the PC12 cells were exposed to 250 *μ*M 6-OHDA, the rates of early apoptosis, late apoptosis, and necrosis were significantly increased by 23.88-fold, 20.84-fold, and 11.51-fold, respectively, compared with the control group. When the PC12 cells were pretreated with FUC, the proportion of apoptotic cells was significantly decreased in a dose-dependent manner (*P* < 0.05, Figure 5(b)). In particular, pretreatment with 5 *μ*M FUC significantly decreased the rates of early apoptosis, late apoptosis, and necrosis by 76.28%, 91.10%, and 53.88%, respectively, compared with the 6-OHDA alone treatment group, indicating that FUC had an obvious inhibitory effect on the apoptosis of PC12 cells induced by 6-OHDA.

The change in mitochondrial membrane potential can largely reflect the activation of cell apoptosis [27]. Therefore, the effect of FUC on the mitochondrial membrane in 6-OHDA-challenged PC12 cells was evaluated via JC-1 staining. The depolarization of the mitochondrial membrane can be associated with an increase in green fluorescence (JC-1 monomer), while the increase in red fluorescence (JC-1 aggregate) reflects the polarization of the mitochondrial membrane [28].  Figure 5(c) reveals that a reduction in red fluorescence and an elevation of green fluorescence were observed in the 6-OHDA alone treatment group compared with the control group. When the cells were pretreated with FUC, the red fluorescence was obviously increased, while the green fluorescence was obviously reduced compared with the 6-OHDA alone treatment group. These results indicated that FUC pretreatment could inhibit 6-OHDA-triggered loss or reduction of mitochondrial membrane potential, contributing to the mitochondrial restoration.

### 3.6. FUC Effectively Protects Zebrafish from 6-OHDA-Induced Neurotoxicity

**Figure 6:**
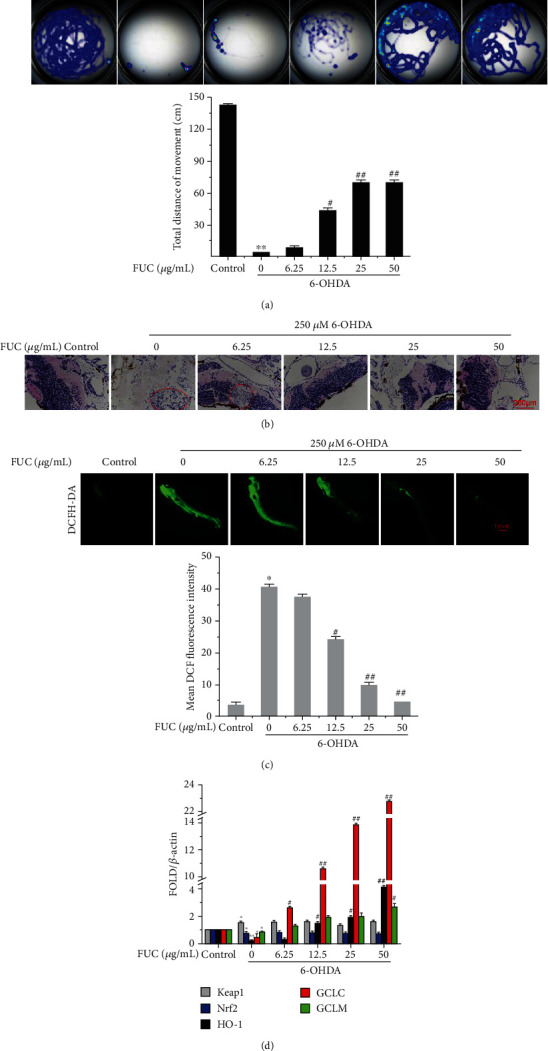
FUC effectively protects zebrafish from 6-OHDA-induced neurotoxicity. (a) FUC improved 6-OHDA-induced movement decreases. Zebrafish larvae at 3 dpf were exposed to FUC with or without 250 *μ*M 6-OHDA for 4 days. Then, larvae were collected for locomotion behavior tests using the Viewpoint Zebrabox system. The total distance traveled in 10 min was calculated. (b) The H&E staining of the zebrafish diencephalon. Scale bar: 200 *μ*m. (c) The effects of FUC on ROS levels in zebrafish larvae. FUC-treated zebrafish larvae were stained with DCFH-DA (10 *μ*M) for 60 min and imaged by a fluorescence microscope at an excitation wavelength of 488 nm and an emission wavelength of 525 nm. Scale bar: 1 mm. (d) The effect of FUC on the expressions of antioxidant genes in 6-OHDA-exposed zebrafish larvae. Total RNA was extracted, and the expressions of Keap1, Nrf2, GCLM, HO-1, and GCLC at the mRNA level were quantified with RT-qPCR and normalized to *β*-actin. ^∗^*P* < 0.05 and ^∗∗^*P* < 0.01 versus the control group. ^#^*P* < 0.05 and ^##^*P* < 0.01 versus the 6-OHDA alone treatment group.

In zebrafish, 6-OHDA can significantly impair DA neurons of zebrafish, leading to a reduction of locomotor behavior [29].  Figure 6(a) shows that 6-OHDA treatment markedly altered the swimming behavior and reduced the total swimming distance of zebrafish larvae by 96.65% compared with the control group, whereas FUC pretreatment reduced such deficit in a dose-dependent manner. Moreover, 50 *μ*g/mL FUC improved 6-OHDA-reduced total movement distance by 14.68-fold compared with the 6-OHDA alone treatment group, indicating that FUC exerted a protective effect against 6-OHDA-induced movement defect.

The H&E staining of the zebrafish diencephalon showed that the zebrafish in the control group had complete brain structure, uniform arrangement of granulosa, and uniform scattering of neuron cell bodies (Figure 6(b)). After the treatment with 6-OHDA, the granulosa in the brain were disorganized, and some cells were broken. With the increased concentration of FUC, the brain tissue structure of zebrafish became tight, the reticular cells in the FUC treatment group were arranged orderly, the neuron cell bodies were evenly distributed, and the proportion of broken cells was reduced in a dose-dependent manner. The 6-OHDA-induced granulosa disorder was reversed when the FUC concentration reached more than 12.5 mg/mL.

Meanwhile, we used the DCFH-DA probe to detect the effects of FUC on the ROS level in zebrafish larvae. 6-OHDA treatment significantly upregulated the ROS level (*P* < 0.05, Figure 6(c)). However, FUC could significantly inhibit 6-OHDA-induced increase of ROS in zebrafish larvae in a dose-dependent manner. When the zebrafish larvae were pretreated with 50 mg/mL FUC, the intensity of green fluorescence was significantly decreased by 89.40% compared with the 6-OHDA alone treatment group.

The effect of FUC on the expressions of Keap1/Nrf2 and downstream antioxidant genes in 6-OHDA-challenged zebrafish larvae was evaluated by RT-qPCR. 6-OHDA treatment significantly increased the level of Keap1 and significantly decreased the expressions of Nrf2, HO-1, GCLC, and GCLM at the mRNA level (*P* < 0.05, Figure 6(d)). With the FUC pretreatment, the expressions of HO-1, GCLC, and GCLM were significantly increased (*P* < 0.05), while the levels of Keap1 and Nrf2 showed no statistically significant differences in 6-OHDA-induced PC12 cells compared with 6-OHDA alone treatment group (*P* > 0.05). For example, pretreatment of 50 *μ*g/mL FUC increased the expressions of HO-1 and GCLC by 20.54-fold and 54.48-fold, respectively, compared with the 6-OHDA alone treatment group.

## 4. Discussion

Oxidative stress is one of the main factors in the pathogenesis of neurodegenerative disorders including PD, resulting in neuronal apoptosis via excessive oxidative stress, mitochondrial dysfunction, and finally cell death [30]. Therefore, it is a palliative and therapeutic strategy to protect DA neurons and effectively prevent the death of DA neurons by antioxidation. Recent studies have found that supplementation of antioxidants in the early stage of PD can effectively improve the neuronal function and suppress the apoptosis of DA neurons to prevent neurodegenerative disorders [31]. As a catecholaminergic neurotoxin, 6-OHDA is a widely used compound to induce PD via oxidative stress [32]. 6-OHDA is autoxidized to release p-quinine, leading to oxidative stress-associated apoptosis [33]. PC12 cells can serve as an optimal PD model as these cells mimic the pathophysiological condition of PD when treated with neurotoxins, such as 6-OHDA [34]. It has been reported that a variety of antioxidants exert potent neuroprotective properties [35, 36]. As a carotenoid compound, FUC possesses neuroprotective, anti-inflammatory, and antioxidant properties [37]. Previous reports have confirmed that FUC reshapes the dynamic balance of ROS and antioxidant levels in vivo and reduces the neurotoxicity induced by *β*-amyloid by increasing the expressions of various antioxidant enzymes [15].

Nrf2-Keap1 is considered to be one of the most critical transduction pathways in regulating the oxidative stress response of cells [38], and it plays an important role in the cellular antioxidant process [39]. Under the normal physiological state, Nrf2 mainly binds to its inhibitor Keap1, exists in the cytoplasm in its inactive state, and is rapidly degraded by ubiquitin-proteasomes to preserve the low transcriptional activity of Nrf2 [40]. However, the consumption of antioxidant enzymes is increased under the condition of excessive ROS production, and the lack of antioxidant scavenging capacity of the organism leads to the imbalance of the oxidation-antioxidant system, thus resulting in oxidative damage to the organism [41]. Therefore, interrupting the formation of Keap1/Nrf2 complex by targeting Keap1 becomes one potential approach to prevent neurodegenerative diseases.

In the present study, we investigated the interaction between FUC and Keap1 at both cell-free molecular and cellular levels. At the cellular level, IP assay revealed the inhibitory effects of FUC on the formation of Keap1/Nrf2 complex. BLI assay demonstrated that FUC could dose-dependently bind to Keap1 protein. The interaction between FUC and Keap1 significantly impaired the Nrf2 binding to Keap1, indicating that the binding site for FUC in Keap1 pocket overlapped that for Nrf2, which was consistent with the molecular docking data. These results demonstrated that Keap1 was a molecular target of FUC.

We further confirmed the binding of FUC to Keap1 through molecular modeling. The protein-protein interplay of Keap1-Nrf2 has been implicated in many neurodegenerative disorders, including Alzheimer's disease and PD [42–44]. However, the X-ray diffraction-based structural information and precise binding mechanism for small molecule compounds to Keap1 protein remain largely unexplored. The computer-assisted simulation has predicted the Keap1 binding sites of small molecule compounds, and the residues Arg^415^ and Tyr^525^ in the Keap1 binding pocket are regarded as molecular targets of many micromolecule suppressors. Londhe et al. [45] have found that binding sites, Arg^483^, Arg^415^, Ser^602^, Tyr^525^ and Tyr^572^, play a significant role in Keap1/Nrf2 complex stability. Ghorab et al. [46] have found that 1,5-dimethyl-2-phenyl-4-(2-phenylquinazolin-4-ylamino)-1,2-dihydropyrazol-3-one (9) binds to Keap1 pocket (Arg^483^, Tyr^525^, and Phe^478^), and then, Keap1 loses its ability to target Nrf2 for ubiquitination and proteasomal degradation. Two novel effective antioxidative tripeptides GWY and QWY [47] have been designed based on 3D-QSAR models, which can improve the stability of Keap1 by interacting with the key residues Arg^415^, Arg^483^, Arg^380^, and Ser^555^ in the active sites. Meanwhile, alanine scanning of both Nrf2 and Keap1 proteins shows that Nrf2 interacts with Keap1 residues Ser^363^, Tyr^380^, Tyr^415^, Tyr^483^, and Ser^508^ primarily through Glu^79^ and Glu^82^, and Nrf2 also binds to Keap1 Tyr^525^ by pi-stacking [48]. In the present study, we investigated the possible binding mechanism of FUC-Keap1 by molecular docking method. The BLI assay revealed that the binding site for FUC in the Keap1 pocket overlapped with that for Nrf2. Using further molecular dynamics, we showed that FUC might form hydrogen bonds with two key residues of Keap1, Arg^415^ and Tyr^525^, which also played a role in the binding of Nrf2. To confirm this hypothesis, two amino acid residues Arg^415^ and Tyr^525^ were substituted with Ala in rhKeap1^R415A+Y525A+^ mutation. As expected, the FortéBio Octet system analysis showed that FUC could not interact with the rhKeap1^R415A+Y525A+^ mutation protein, indicating that Arg^415^ and Tyr^525^ played a critical role in FUC-Keap1 interactions. Therefore, the results of this study clarified the antioxidant targets of FUC and provided the important structural understanding of the amino residue sites for further design of Keap1 inhibitors as antioxidative agents.

Nrf2 dissociates from Keap1 and translocates to the nucleus, where it binds to the Maf protein to form a heterodimer and then binds to ARE to regulate the transcriptional activity of phase II metabolic enzymes [25, 26]. Previous investigations have shown that 6-OHDA-exposed cell line increases Keap1 and decreases Nrf2 expression [49]. Consistent with previous works, our present study revealed that 6-OHDA induced a remarkable reduction of Nrf2 expression and upregulation of Keap1 protein, but FUC pretreatment did not affect the expressions of Keap1 protein and the total mRNA level of Nrf2 in 6-OHDA-induced PC12 cells and zebrafish. However, the inhibitory effects of FUC on Keap1 resulted in activated Nrf2/Keap1-ARE pathway, increased the accumulation of nuclear Nrf2 protein, and induced ARE-Luc reporter activity in 6-OHDA-stimulated PC12 cells, evidenced by a dose-dependent enhancement in the expressions of HO-1, GCLC, and GCLM, as well as the reduction of ROS and cell death.

Mitochondrial dysfunction is a pathogenic event, which is closely related to oxidative stress that contributes to PD pathogenesis. 6-OHDA affects mitochondrial membrane function by suppressing the electron transport chain [50]. Consistent with previous works, our study showed that 6-OHDA triggered a dramatic reduction in mitochondrial membrane potential. However, the apoptosis of 6-OHDA-exposed PC12 cells was blocked by FUC pretreatment, and the underlying mechanism might be through protecting the permeability of the mitochondrial membrane. In vivo, FUC pretreatment significantly increased the motor ability and decreased the ROS production in the 6-OHDA-exposed zebrafish. Brain histological changes in the 6-OHDA-exposed zebrafish were also suppressed by FUC pretreatment. These findings indicated that FUC could be developed as a therapeutic agent in the management of neurodegenerative disorders.

## 5. Conclusions

**Scheme 1:**
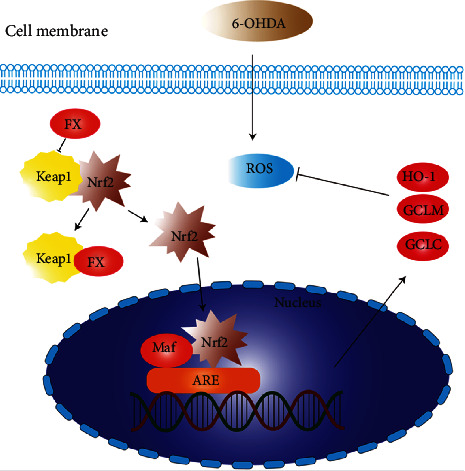
Proposed model of signaling pathway involved in FUC prevented 6-OHDA-induced ROS.

Collectively, our data revealed that Keap1 was the target of FUC and could block the formation of Keap1/Nrf2 complex. Moreover, FUC could directly bind to Keap1, reversed the inhibits of 6-OHDA on Keap1/Nrf2-ARE, increased the expressions of downstream antioxidant enzymes, and decreased the levels of ROS and cell apoptosis in 6-OHDA-exposed PC12 cells (Scheme 1). Arg^415^ and Tyr^525^ in the Keap1 protein played an important role in the interaction between FUC and Keap1 via two hydrogen bonds. In vivo, FUC improved motorability of zebrafish larvae and protected the brain injury induced by 6-OHDA. This study suggested that as a new Keap1 inhibitor, FUC might protect nerve cells from oxidative damage by targeting Keap1.

## Data Availability

The datasets generated and/or analyzed during the present study are available from the corresponding author on reasonable request.

## Supplementary Materials

Supplementary Materials Dataset 1: pET28a-Keap1-Mut DNA sequencing. Dataset 2: Keap1-Mut open reading frame translation sequencing. Dataset 3: Keap1 open reading frame translation sequencing (PDB ID: 4IFN, Homo sapiens).
