# Supplementary material for: Fucoxanthin Prevents 6-OHDA-Induced Neurotoxicity by Targeting Keap1
Source: Oxid Med Cell Longev. 2021 Mar 11;2021:6688708. doi: 10.1155/2021/6688708 (PMC7972864; doi:10.1155/2021/6688708)
Supplement: Supplementary Materials — Dataset 1: pET28a-Keap1-Mut DNA sequencing. Dataset 2: Keap1-Mut open reading frame translation sequencing. Dataset 3: Keap1 open reading frame translation sequencing (PDB ID: 4IFN, Homo sapiens). [file 6688708.f1.docx]

# Fucoxanthin prevents 6-OHDA-induced neurotoxicity by targeting Keap1

Wei Wu^1*^, Hui Han^1*^, Jingwangwei Liu^1^, Min Tang^1^, Xiaoyu Wu^1^, Xiaojun Cao^1^, Tiantian Zhao^1^, Yujia Lu^1^, Tingting Niu^1^, Juanjuan Chen^1^, Haimin Chen^1#^

^1^ State Key Laboratory for Managing Biotic and Chemical Threats to the Quality and Safety of Agro-products, Ningbo University, Ningbo, Zhejiang 315211, China^1^ Department,

Correspondence should be addressed to Haimin Chen; chenhaimin@nbu.edu.cn (H. C.)

**pET28a-KEAP1-Mut DNA sequencing**

CATATGGCACCGAAAGTTGGTCGTCTGATTTATACCGCAGGCGGTTATTTTCGTCAGAGCCTGAGCTATCTGGAAGCATATAATCCGAGTGATGGCACCTGGCTGCGTCTGGCCGATCTGCAGGTTCCGCGTAGCGGTCTGGCAGGTTGTGTTGTTGGTGGTCTGCTGTATGCAGTTGGTGGCCGTAATAATAGTCCGGATGGTAATACCGATAGCAGCGCACTGGATTGTTATAACCCGATGACCAATCAGTGGTCACCGTGTGCACCGATGAGCGTTCCTCGT**AAT**GCAATTGGTGTTGGTGTTATTGATGGCCATATTTATGCCGTTGGCGGTAGCCATGGTTGTATTCATCATAATAGCGTGGAACGTTATGAACCGGAACGTGATGAATGGCATCTGGTGGCACCGATGCTGACCCGTCGTATTGGTGTGGGCGTTGCAGTTCTGAATCGTCTGTTATATGCCGTAGGTGGTTTTGATGGTACAAATCGTCTGAATAGCGCAGAATGTTATTATCCGGAACGCAACGAATGGCGTATGATTACCGCAATGAATACCATTCGTAGCGGTGCCGGTGTTTGTGTTCTGCATAATTGTATTTATGCAGCCGGT**GGT**GCAGATGGTCAGGATCAGCTGAATTCTGTTGAACGCTATGATGTTGAAACCGAAACCTGGACCTTTGTTGCTCCGATGAAACATCGTCGTAGTGCACTGGGTATTACCGTTCATCAGGGTCGTATTTATGTGTTAGGTGGCTATGATGGTCATACCTTTCTGGATAGCGTTGAGTGCTATGATCCGGATACCGATACTTGGAGCGAAGTTACCCGTATGACCAGCGGTCGTTCAGGCGTTGGTGTTGCAGTTACCTAACTCGAG

**KEAP1-Mut open reading frame translation sequencing**

MAPKVGRLIYTAGGYFRQSLSYLEAYNPSDGTWLRLADLQVPRSGLAGCVVGGLLYAVGGRNNSPDGNTDSSALDCYNPMTNQWSPCAPMSVPRN**A**IGVGVIDGHIYAVGGSHGCIHHNSVERYEPERDEWHLVAPMLTRRIGVGVAVLNRLLYAVGGFDGTNRLNSAECYYPERNEWRMITAMNTIRSGAGVCVLHNCIYAAGG**A**DGQDQLNSVERYDVETETWTFVAPMKHRRSALGITVHQGRIYVLGGYDGHTFLDSVECYDPDTDTWSEVTRMTSGRSGVGVAVT

**KEAP1 open reading frame translation sequencing (PDB ID: 4IFN, Homo sapiens)**

SSAPKVGRLIYTAGGYFRQSLSYLEAYNPSDGTWLRLADLQVPRSGLAGCVVGGLLYAVGGRNNSPDGNTDSSALDCYNPMTNQWSPCAPMSVPRN**R**IGVGVIDGHIYAVGGSHGCIHHNSVERYEPERDEWHLVAPMLTRRIGVGVAVLNRLLYAVGGFDGTNRLNSAECYYPERNEWRMITAMNTIRSGAGVCVLHNCIYAAGG**Y**DGQDQLNSVERYDVETETWTFVAPMKHRRSALGITVHQGRIYVLGGYDGHTFLDSVECYDPDTDTWSEVTRMTSGRSGVGVAVT
